# Fate of Ingested *Clostridium difficile* Spores in Mice

**DOI:** 10.1371/journal.pone.0072620

**Published:** 2013-08-30

**Authors:** Amber Howerton, Manomita Patra, Ernesto Abel-Santos

**Affiliations:** Department of Chemistry, University of Nevada - Las Vegas, Las Vegas, Nevada, United States of America; Loyola University Medical Center, United States of America

## Abstract

*Clostridium difficile* infection (CDI) is a leading cause of antibiotic-associated diarrhea, a major nosocomial complication. The infective form of *C. difficile* is the spore, a dormant and resistant structure that forms under stress. Although spore germination is the first committed step in CDI onset, the temporal and spatial distribution of ingested *C. difficile* spores is not clearly understood. We recently reported that CamSA, a synthetic bile salt analog, inhibits *C. difficile* spore germination *in vitro* and *in vivo*. In this study, we took advantage of the anti-germination activity of bile salts to determine the fate of ingested *C. difficile* spores. We tested four different bile salts for efficacy in preventing CDI. Since CamSA was the only anti-germinant tested able to prevent signs of CDI, we characterized CamSa’s *in vitro* stability, distribution, and cytotoxicity. We report that CamSA is stable to simulated gastrointestinal (GI) environments, but will be degraded by members of the natural microbiota found in a healthy gut. Our data suggest that CamSA will not be systemically available, but instead will be localized to the GI tract. Since *in vitro* pharmacological parameters were acceptable, CamSA was used to probe the mouse model of CDI. By varying the timing of CamSA dosage, we estimated that *C. difficile* spores germinated and established infection less than 10 hours after ingestion. We also showed that ingested *C. difficile* spores rapidly transited through the GI tract and accumulated in the colon and cecum of CamSA-treated mice. From there, *C. difficile* spores were slowly shed over a 96-hour period. To our knowledge, this is the first report of using molecular probes to obtain disease progression information for *C. difficile* infection.

## Introduction

*Clostridium difficile* infection (CDI) is the major identifiable cause of antibiotic-associated diarrhea in hospitals [1]. In the US alone, CDI develops in over 500,000 patients with up to 20,000 deaths per year [2]. The yearly health care burden has been estimated to be greater than $3 billion.

The infective agent of CDI is the *C. difficile* spore, a hardy structure formed under nutrient deprivation [3]. In a healthy gut, indigenous microbes form a protective barrier against *C. difficile* colonization of the gastrointestinal (GI) tract, but this protective function can be weakened by antibiotic therapy [4]. Under these favorable conditions, *C. difficile* spores interact with small molecule germinants, triggering a series of events committing the spore to germinate into toxin producing bacteria [5].

Since spore germination is the first committed step in CDI, understanding the behavior of spores in the GI tract of the host is a necessary first step in infection control [1]. Taurocholate, a natural bile salt, and glycine, an amino acid, were shown to activate *C. difficile* spore germination [6]. We have reported that *C. difficile* spores bind taurocholate and glycine through a complex mechanism [7]. Using kinetic analysis, we showed that unknown receptor homo- and heterocomplexes are formed. Others and we also showed that chenodeoxycholate, another natural bile salt, is a competitive inhibitor of *C. difficile* spore germination [7], [8], [9], [10]. These findings strongly implicate the presence of unidentified proteinaceous germination receptor(s) that *C. difficile* uses to bind small molecules to activate spore germination.

Analogs of taurocholate and glycine were used as chemical probes to determine structure activity relationships for germinant binding and activation of germination of *C. difficile* spores *in vitro* [8]. The putative germination machinery of *C. difficile* seems to contain unique binding sites for alkyl, aromatic, and basic amino acids as co-germinants whereas the binding region for bile salts is restricted to taurocholate analogs [8]. We reported that a *meta*-benzene sulfonic acid derivative of taurocholate, CamSA, is a strong competitive inhibitor of taurocholate-mediated *C. difficile* spore germination *in vitro*. Even more, a single 50 mg/kg dose of CamSA prevented CDI in mice without any observable toxicity [11]. Our results support a mechanism whereby the anti-germination effect of CamSA is responsible for preventing CDI signs.

Although the germination of *C. difficile* spores has been studied *in vitro*, the *in vivo* fate of ingested spores is not clear [1]. Determining the timing of ingested spore germination will allow assessing the time window when patients are at risk of developing CDI. Furthermore, determining the transit time of ingested spores through the GI tract will allow defining whether ingested spores contribute to CDI relapse.

Understanding the fate of ingested spores has been hampered by the rapid CDI progression from spore challenge to clinical endpoint in the hamster model of CDI [12]. This is further complicated by the ability of *C. difficile* vegetative cells to re-sporulate in the intestine of the animal host [13]. Indeed, previous works have not been able to distinguish between ingested spores and spores formed in the gut of infected animals [14].

In the current study, we tested the ability of four bile salt analogs as *in vivo* inhibitors of *C. difficile* spore germination. Since CamSA had the best biological activity, we further characterized CamSA’s stability, distribution and cytotoxicity *in vitro*. Finally, we used CamSA as a probe to estimate the transit time of ingested *C. difficile* spores and the timing of CDI onset in mice. With this information we proposed a model that describes the spatial and temporal fate of ingested *C. difficile* spores.

## Results

### CamSA had no Observable Adverse Effects on Mice

To determine the acute toxicity of CamSA to mice, we used the fixed dose procedure [15]. No physical adverse effects or weight loss were observed when CamSA was administered for three consecutive days at doses up to saturating 300 mg/kg (Fig. S1). A 300 mg/kg dose of chenodeoxycholate caused immediate death, probably due to observed precipitation of chenodeoxycholate upon interaction with mouse saliva and gastric juice. Chenodeoxycholate at 50 mg/kg did not cause any observable side effects.

### Prevention of CDI by Bile Salt Analogs

**Figure 1:**
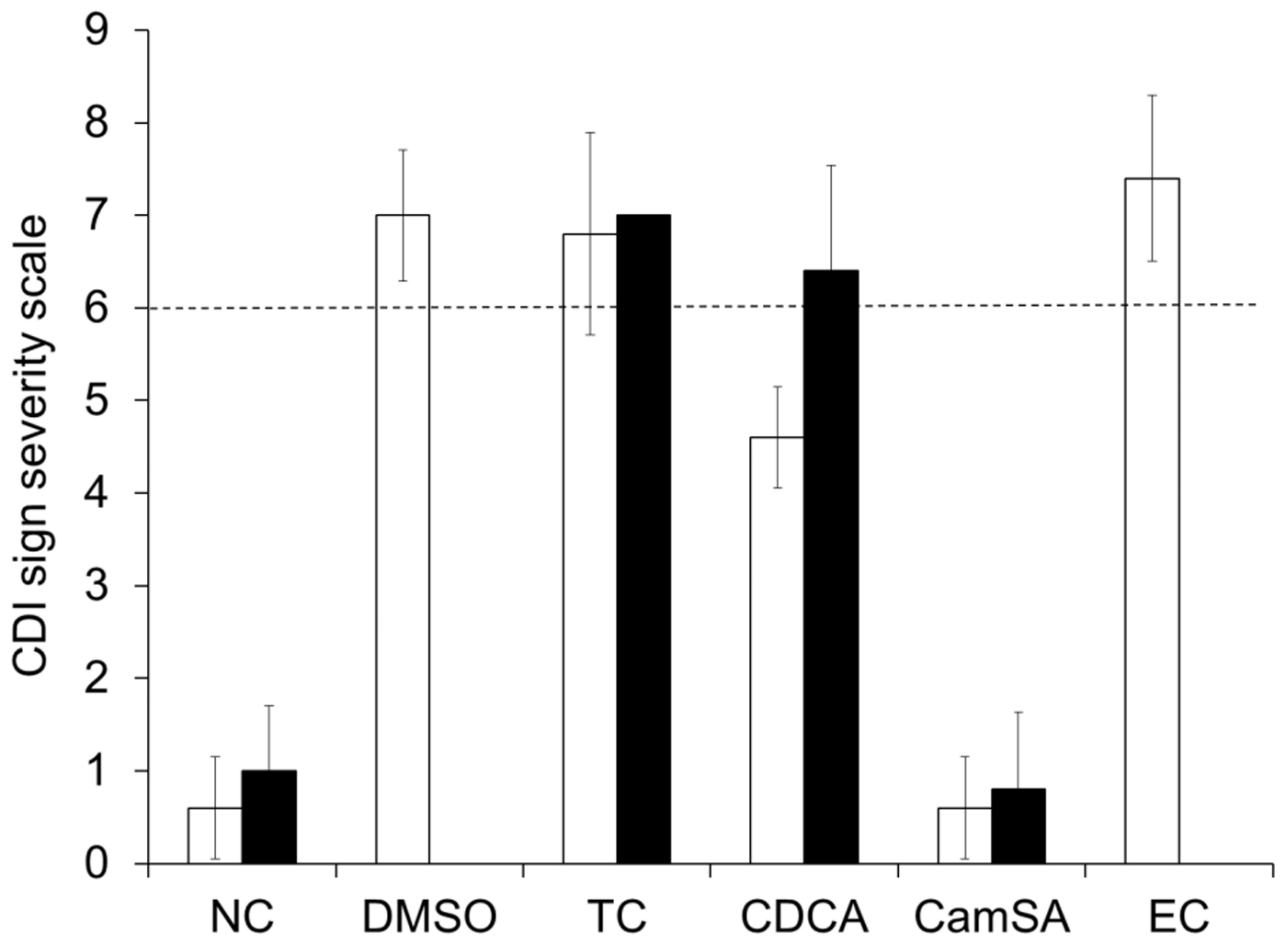
CamSA protects mice from CDI. Comparison of CDI sign severity after 48 hours (white bars) and 72 hours (black bars) of animals challenged with *C. difficile* spores and treated with DMSO, 300 mg/kg taurocholate (TC), 50 mg/kg chenodeoxycholate (CDCA), 50 mg/kg CamSA, or 300 mg/kg ethyl cholate (EC). Non-challenged (NC) animals were used as controls. Clinical endpoint was set as >6 in the CDI sign severity scale (dashed line). None of the animals in the DMSO and EC groups survived to 72 hours post-challenged. Standard deviations represent at least five independent measures.

As previously reported, when mice were challenged with 10^8^ CFU of *C. difficile* spores, severe CDI signs developed and all animals reached clinical endpoint by 48 hours post-challenge [16]. The large (10^8^ CFUs) inoculum of spores ensured synchronized CDI onset and fast CDI sign progression. Mice treated with up to 300 mg/kg taurocholate or ethyl cholate also developed severe CDI and signs were undistinguishable from control DMSO-treated animals (Fig. 1 and Fig. S2). Mice treated with 50 mg/kg chenodeoxycholate developed moderate to severe signs of CDI, but onset was delayed by 24 hours (Fig. S3). In contrast, all animals treated with 50 or 300 mg/kg CamSA showed no sign of CDI and were undistinguishable from non-challenged animals [11]. All asymptomatic animals remained free of CDI signs for at least 14 days post-challenge.

### Stability of CamSA

CamSA is a taurocholate analog with an amide bond linking cholic acid to *meta*-aminobenzene sulfonic acid. To be effective, CamSA must survive the changing environments of the GI tract. To test for stability, CamSA was incubated in artificial gastric juice and intestinal juice. No degradation of CamSA was evident even after 24 hours incubation under both conditions (data not shown).

**Figure 2:**
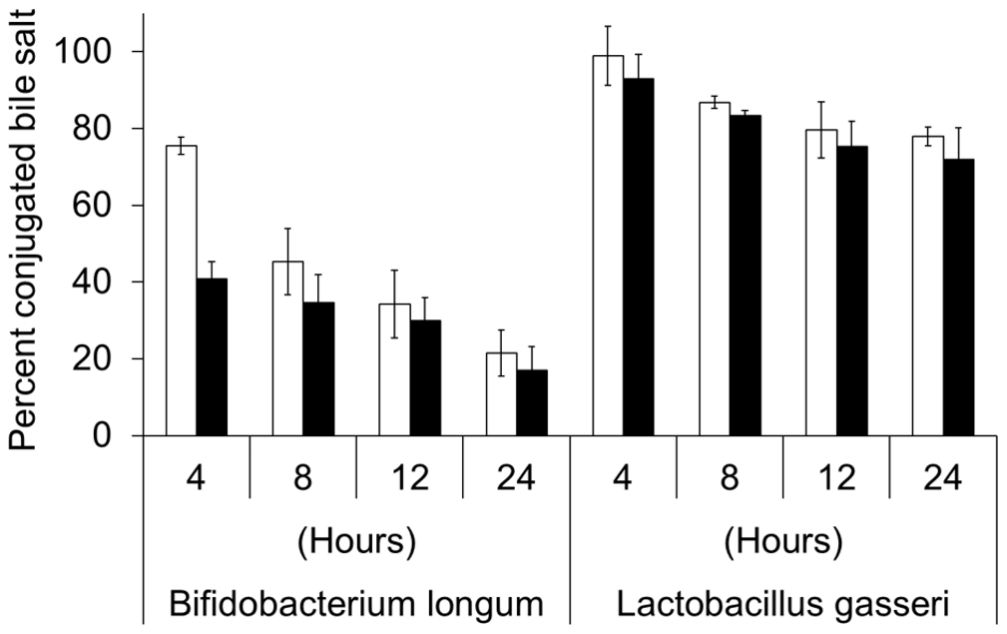
Stability of CamSA and taurocholate towards bile salt hydrolases. CamSA (white bar) and taurocholate (black bars) were incubated with cultures of *B. longum* or *L gasseri*. Percent conjugated bile salts were derived by dividing the intensity of TLC spots obtained at different times by the intensity of the TLC spot obtained at the beginning of incubation (time 0). Time 0 was set at 100% and is not shown for clarity. Standard deviations represent at least five independent measures.

Bacterial bile salt hydrolases (BSHs) deconjugate primary and secondary bile salts [17]. *B. longum* and *L. gasseri* are two intestinal bacteria commonly used as test strains for BSH production. After incubation with a culture of *B. longum* for 24 hours, CamSA and taurocholate are both hydrolyzed to cholic acid at similar rates (Fig. 2). CamSA and taurocholate are less sensitive to degradation by BSHs secreted by *L. gasseri.* Less than 30% of either CamSA or taurocholate was hydrolyzed to cholic acid after 24 hours (Fig. 2). *E. coli* does not produce BSH and both CamSA and taurocholate were stable after 24 hour incubation with *E. coli* cultures (data not shown). CamSA was not degraded in growth medium alone.

### Caco-2 Permeability of CamSA

To prevent *C. difficile* spores from germinating, CamSA needs to be retained in the intestinal lumen. Caco-2 monolayers serves as an *in vitro* surrogate assay for intestinal permeability, absorption, and metabolism [18]. CamSA was studied in a Caco-2 permeability assay and displayed an apical to basolateral apparent permeability coefficient (P_app_) of <10^−6 ^cm/s and basolateral to apical P_app_ of 10.9×10^−6 ^cm/s. The efflux ratio suggests that CamSA is a substrate for active transport (Table S1). In both assays, CamSA was recovered at 100% indicating low binding, accumulation, and metabolism by Caco-2 cells.

### Effect of CamSA on Bacterial Growth

*E. coli*, *B. longum*, and *L. gasseri* are indigenous mammalian gut bacteria and are continuously exposed to bile salts [17]. As expected, growth of these bacteria was unaffected by the presence of CamSA in the growth medium. *C. difficile* cells also grew normally in the presence of CamSA (Fig. S4).

### Cytotoxicity of CamSA

**Figure 3:**
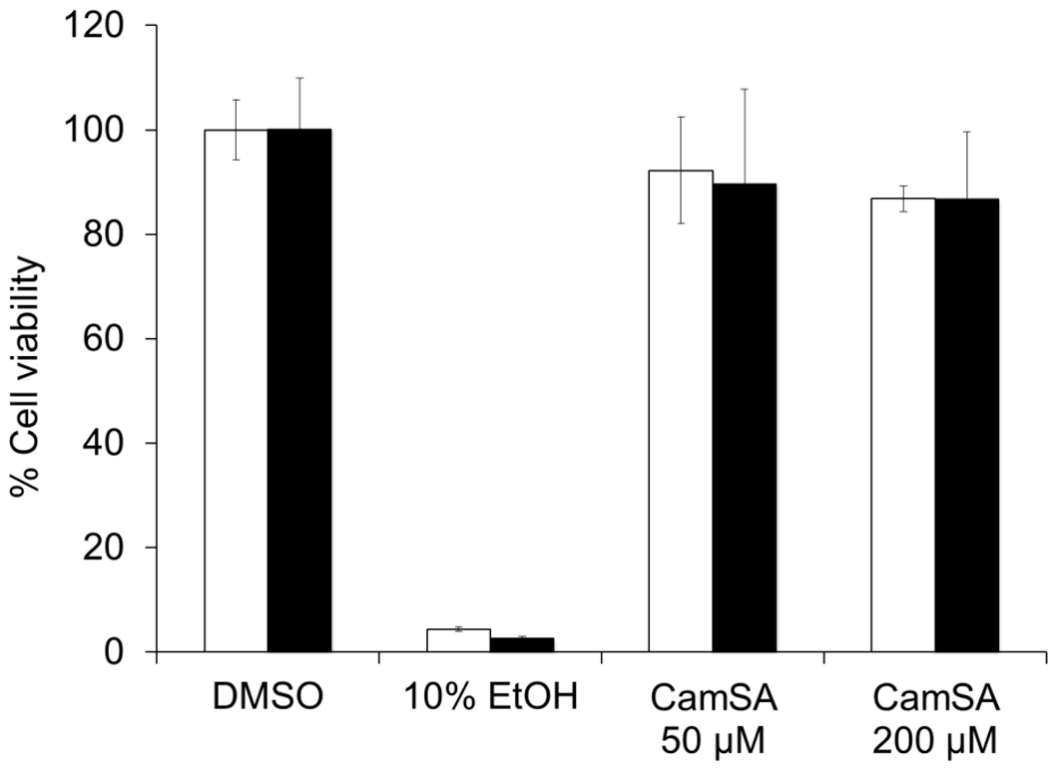
Cytotoxicity of CamSA. Vero cells (white bars) or Caco-2 cells (black bars) were incubated overnight with 10% DMSO, 10% EtOH, 50 µM CamSA or 200 µM CamSA. Cell viability was determined with the CellTiter Glo viability kit. The luminescence signal from DMSO-treated cells was undistinguishable from untreated cells and was set as 100% cell viability. Percent survival for other conditions was calculated relative to untreated cells. Error bars represent standard deviations from at least five independent measurements.

Cell viability was qualitatively determined by visual observation of rounded/detached cells and trypan blue staining. CamSA-treated Vero, Caco-2, and macrophage cells appeared healthy and were undistinguishable from DMSO-treated cells (Fig. S5). Cell viability was also quantitatively determined by ATP production. Vero and Caco-2 cells treated with 50 or 200 µM CamSA produced ATP at similar levels to healthy control cells (Fig. 3).

### CamSA Protection of Vero and Caco-2 Cells

**Figure 4:**
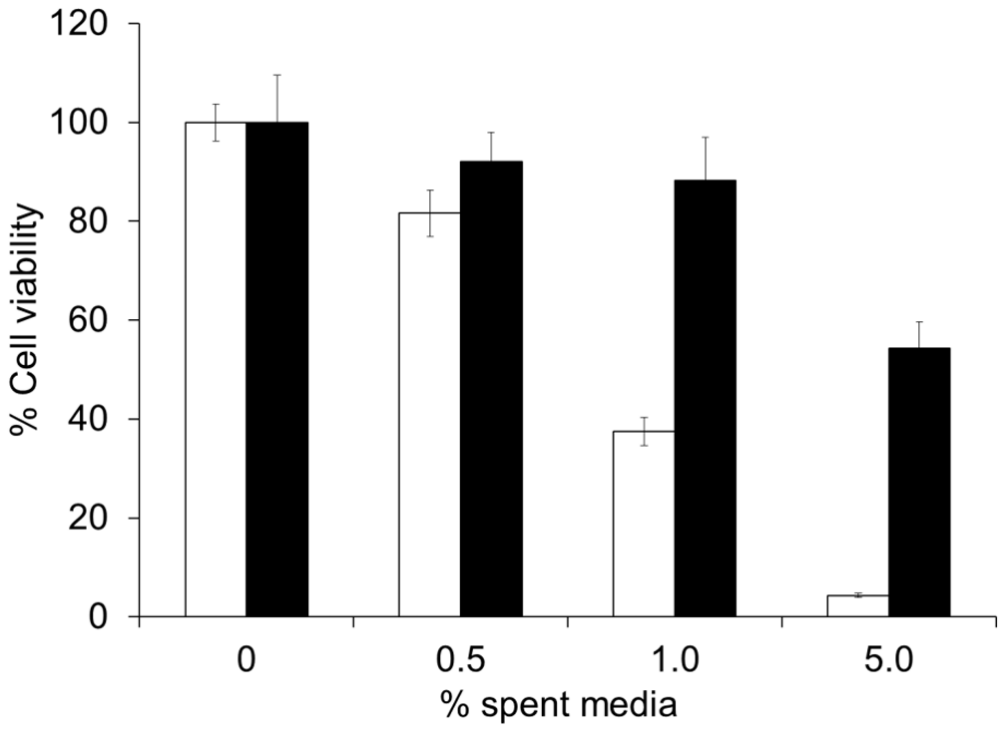
Inhibition of *C. difficile* toxin production by CamSA treatment. *C. difficile* spores were incubated overnight in media containing 0 µM CamSA (white bars) or 200 µM CamSA (black bars). The resulting spent media were added to Vero cell cultures and incubated for 24 hours. Cell viability was determined with the CellTiter Glo viability kit. The luminescence signal from untreated cells was set as 100% cell viability. Percent survival for other conditions was calculated relative to untreated cells. Error bars represent standard deviations from at least five independent measurements.

Spent media from outgrowing *C. difficile* spores killed Vero cells in a dose-dependent manner (Fig. 4). These data are consistent with previous reports indicating that vegetative *C. difficile* secretes cell-killing toxins during growth [19]. When *C. difficile* spores were incubated in medium containing 200 µM CamSA, bacterial growth was reduced but not eliminated. As expected, spent media from CamSA-treated bacterial cultures were less effective at killing epithelial cells. Similar results were observed for Caco-2 cell cultures (data not shown).

### Timing of CDI Onset

**Figure 5:**
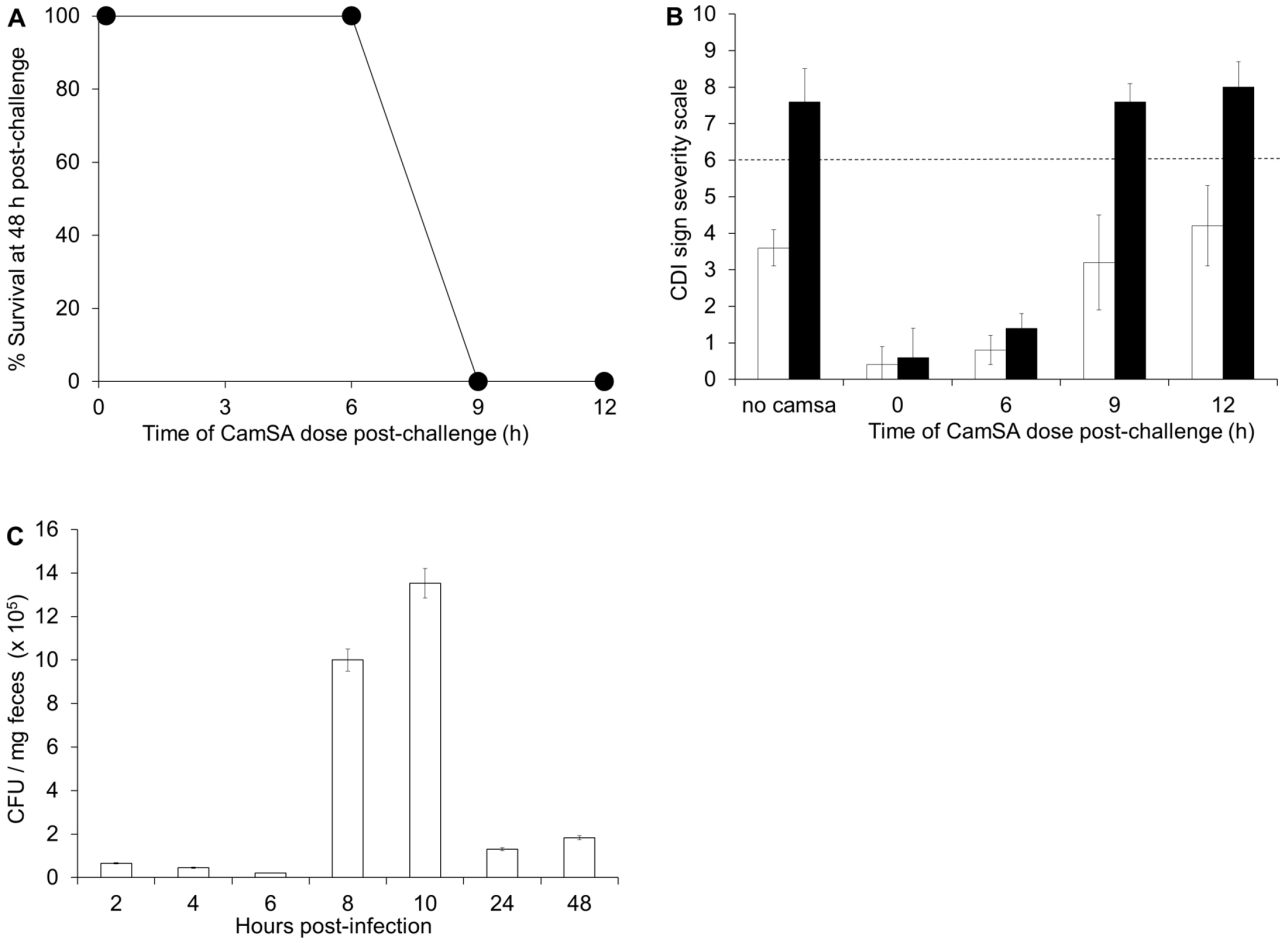
CDI is established between 6 and 9 hours post-infection. (A) Survival of infected mice at 48 hours after challenge with *C. difficile* spores. Mice were treated with 300 mg/kg CamSA at 0, 6, 9, or 12 hours post-challenge. (B) Comparison of CDI severity after 24 hours (white bars) and 48 hours (black bars) for animals challenged with *C. difficile* spores and treated with 300 mg/kg CamSA at 0, 6, 9, or 12 hours post-challenge. Clinical endpoint was set as >6 in the CDI sign severity scale (dashed line). (C) *C. difficile* vegetative cell count in feces of untreated, diseased animals. Feces were collected from cages housing five untreated mice challenged with *C. difficile* spores. Open bars represent *C. difficile* vegetative cells. The amount of *C. difficile* spores excreted by untreated animals was negligible (<10% of vegetative cell counts). Standard deviations represent at least five independent measures. Recovered CFU and recovered spores represent mean values from pools of five animals.

To determine the onset of CDI in mice, animals were challenged with *C. difficile* spores and treated with 300 mg/kg CamSA between 0 and 12 hours post-challenge. All animals treated with CamSA up to 6 hours post-challenge were fully protected from CDI. In contrast, all animals treated with CamSA at 9 or 12 hours post-challenge developed severe CDI undistinguishable from untreated mice and reached the clinical endpoint 48 hours post infection (Figs. 5A and 5B).

Similar to previous reports, GI contents from animals with CDI signs contained almost exclusively *C. difficile* vegetative cells [13]. These animals started to excrete large amounts (>10×10^5^ CFUs) of vegetative cells reaching a maximum between 8 and 10 hours post spore challenge (Fig. 5C). Although some *C. difficile* spores were excreted by diseased animals, the amounts were negligible (<10% of vegetative CFUs) compared to the high amount of excreted vegetative cells.

### Recovery of *C. difficile* Cells and Spores from Intestines and Feces of CamSA-treated Mice

**Figure 6:**
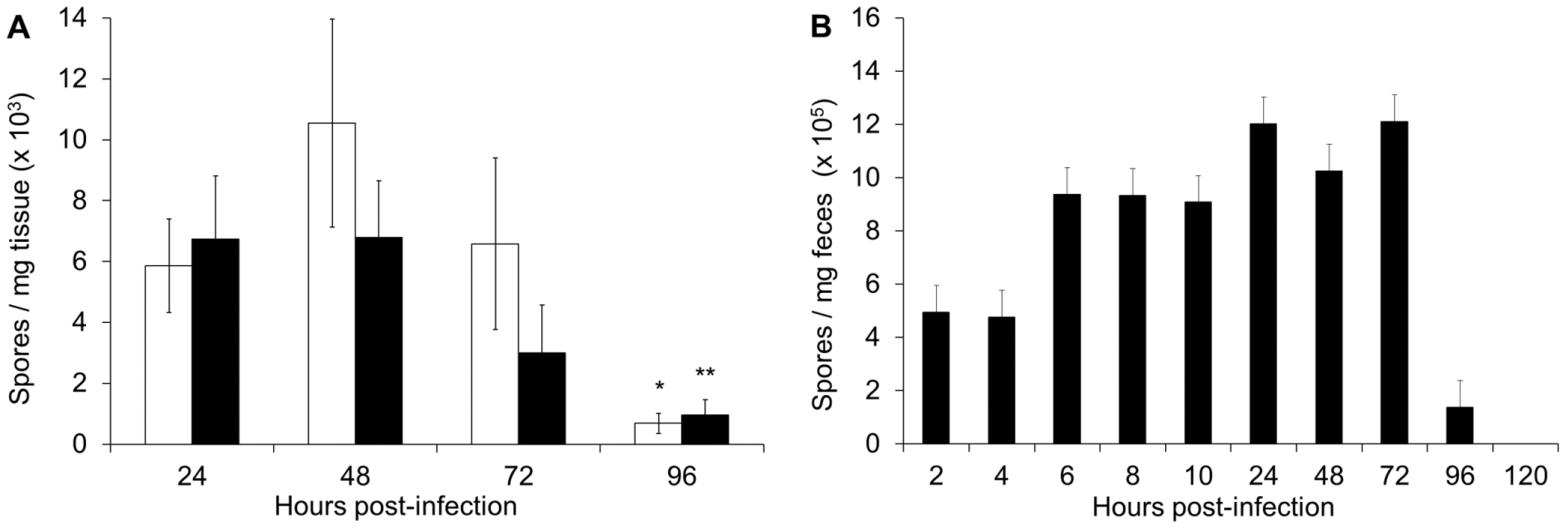
*C. difficile* spores accumulate in the cecum, colon, and feces of CamSA-treated animals. (A) Amount of *C. difficile* spores recovered at different time points following spore challenge from the cecum (white bars) and colon (black bars) of mice treated with 50 mg/kg CamSA. Student’s unpaired *t*-test was used to determine the significance of difference of means. *indicates recovered spores significantly below 72 hour levels (*P* = 0.019; Student's *t*-test). **indicates recovered spores significantly below 72 hour levels (*P* = 0.049; Student's *t*-test). (B) Feces were collected from cages housing five mice challenged with *C. difficile* spores and treated with 50 mg/kg CamSA. Closed bars represent *C. difficile* spores. The amount of *C. difficile* vegetative cells in CamSA-treated animals was negligible (<10% compared to spore counts). Standard deviations represent at least five independent measures. Recovered CFU and recovered spores represent mean values from a pool of five animals.

Similar to the hamster CDI model [13], ingested *C. difficile* spores narrowly localized to the cecum and colon of CamSA treated mice at every time point tested. A negligible amount of *C. difficile* was discovered in the small intestine and stomach (Fig. S6). *C. difficile* spores remained in the cecum and colon for 72 hours after spore challenge (Fig. 6A). By 96 hours, the amount of spores recovered from the cecum and colon of CamSA treated animals decreased almost tenfold, from greater than 12×10^5^ to less than 2×10^5^ CFUs.

Consistent with the results from intestinal content, the feces of CamSA-treated animals contained almost exclusively spores (Fig. 6B). In these animals, excretion of ingested *C. difficile* spores started 2 hours post-challenge and continued until at least 96 hours post-challenge. In fact, by 120 hours post-challenge, the sum of excreted *C. difficile* spores was quantitatively identical to the number of spores given by gavage.

## Discussion

Germination of *C. difficile* spores is believed to be the first step in establishing CDI [1]. However, determining the fate of ingested *C. difficile* spores is challenging since spores can germinate and the resulting cells can then re-sporulate perpetuating a cycle of disease. Anti-germinants prevent CDI by effectively freezing ingested spores in their dormant state. We took advantage of this anti-germinant property to study the temporal and spatial distribution of ingested *C. difficile* spores.

Following our success using the anti-germination properties of CamSA to protect mice from CDI, we tested taurocholate (a natural germination enhancer), chenodeoxycholate (a natural germination inhibitor), and ethylcholate (a commercially available germination inhibitor) as prophylactics of CDI. Ethyl cholate inhibits *C. difficile* spore germination with a half maximal inhibitory concentration (IC_50_) of 8.2 µM, seven-fold more potent than CamSA *in vitro* (data not shown). Chenodeoxycholate, on the other hand, inhibits *C. difficile* spore germination with an IC_50_ of 235 µM, approximately five-fold less potent than CamSA [20].

As previously reported, CamSA was a prophylactic of murine CDI [11]. In this study, we were able to prevent CDI with saturating concentrations of CamSA without any overt toxic effects. This is consistent with the low toxicity observed for taurocholate and cholate [21]. In fact, CamSA had anti-CDI activity at concentrations that were at least six times lower than its toxic threshold. Furthermore, CamSA-treated animals did not show signs of CDI even 14 days post-challenge. At 300 mg/kg concentration, chenodeoxycholate showed acute toxicity probably due to low solubility in biological fluids. At lower concentrations, chenodeoxycholate was not able to prevent CDI. Animals treated with 50 mg/kg chenodeoxycholate show the same CDI sign patterns as animals treated with 5 mg/kg CamSA [11]. Another potent inhibitor of *C. difficile* spore germination *in vitro*, ethylcholate, did not protect mice from CDI even at 300 mg/kg. Similarly, taurocholate, a germinant, was unable prevent CDI. CamSA was the only compound tested that was effective in preventing CDI. Hence, we further characterized CamSA’s *in vitro* pharmacological properties.

To be effective as *in vivo* probes, anti-germination compounds must be stable to the variable GI tract environments. CamSA is stable to all tested GI tract microenvironments except incubation with the BSH-producing bifidobacteria. Antibiotics disrupt the normal microflora dynamics of the gut allowing for outgrowth of *C. difficile* [22]. A recent study shows that antibiotic cocktails shift the normal murine gut bacterial population to a preponderance of *Lactobacilli* [23]. Since BSHs produced by *Lactobacilli* were not effective in hydrolyzing CamSA, CamSA should remain stable in the gut of antibiotic-treated mammals.

Since CDI is an intestinal infection, anti-germination compounds require low oral bioavailability and high GI tract stability for maximum efficacy. *In vitro* assays suggest that CamSA will be retained in the intestinal tract of mammals (Table S1). The efflux ratio of CamSA suggests that it is a substrate for Pgp [24]. Hence, the efficacy of CamSA might be in part due to retention in the GI tract due to Pgp mediated excretion of the drug back into the intestinal lumen. The poor bioavailability of CamSA will also reduce toxic effects to other organs since CamSA is unlikely to circulate outside of the intestinal lumen. Tox-ADME analyses also suggest low metabolism of CamSA by intestinal epithelial cells and further supports that CamSA will remain stable in the intestinal lumen.

Because the natural microbiota is key to resist *C. difficile* infection, anti-germination compounds should not damage this natural barrier [22]. Indeed, CamSA did not affect growth of commensal bacteria, *B. longum*, *L. gasseri,* or *E. coli.* Although CamSA inhibits spore germination *in vitro*, it does not affect *C. difficile* vegetative growth (Fig. S4). These data support the proposed mechanism that CamSA inhibits spore germination and does not act as an antibacterial agent. Anti-germination compounds should also show low toxicity toward mammalian cells. Indeed, CamSA did not affect the viability of two different mammalian epithelial cell lines nor did it affect macrophage immune cells.

Since toxin is only secreted by metabolically active *C. difficile* cells, halting germination should result in less toxin production. Tissue culture protection experiments rely on decreasing the accumulation of toxins secreted into media during *C. difficile* vegetative growth [19]. CamSA inhibits *C. difficile* spore germination *in vitro* and hence protects mammalian cells by reducing the number of toxin-producing bacteria.

Previous works have not been able to distinguish between ingested *C. difficile* spores and spores that were produced after colonization of the host’s GI tract. Indeed, enumeration of *C. difficile* in feces and GI content from infected hamsters yields mixtures of vegetative cells and spores [13], [14]. Furthermore, enumerated spore loads were higher than the original inoculum. These data suggest that ingested *C. difficile* spores germinated and re-sporulated during colonization of the hamster gut. Due to the fast progression of CDI in untreated mice, we could only determine bacterial loads for the first 48 hours after challenge. Even then, we could not distinguish whether spores recovered from diseased animals came from the original inoculum or from re-sporulation in the intestines.

Since CamSA shows favorable *in vitro* pharmacological properties and can block *C. difficile* spore germination *in vivo*, we were able to follow the fate of ingested *C. difficile* spores without interference from germination and/or re-sporulation. CamSA was effective in preventing CDI when administered up to six hours following spore challenge. In contrast, CamSA was ineffective when administered nine hours post-challenge, even at the highest concentration tested. This narrow three-hour window correlates *C. difficile* spore germination with maximum *C. difficile* vegetative cell shedding in symptomatic mice. These data suggest that a fraction of germinated *C. difficile* cells are excreted soon after germination, while the remaining *C. difficile* vegetative cells lead to CDI onset.

Human CDI shows more heterogeneous symptoms and longer disease progression than rodent models [12]. CamSA treatment allowed us to observe the behavior of ungerminated spores for a period extended beyond the normal clinical endpoint of CDI-diseased mice. Ingested *C. difficile* spores were quantitatively recovered from feces, cecum, and colon contents of CamSA-treated mice. Interestingly, ingested *C. difficile* spores started to be shed soon after challenge, but part of the population remained in the lower GI for up to four days.

The mechanism of dormant *C. difficile* spore accumulation in the lower intestine is not understood, but suggests that ingested *C. difficile* spores can form a transitory reservoir that is slowly released from the lower intestine. A possibility is that a small fraction of *C. difficile* spores enter a superdormant state that helps them to be retained in the intestine [25]. Although the amount of unattached spores in the intestines is small, it is tempting to speculate that these spore reservoirs serve as a focal point for CDI relapse.

**Figure 7:**
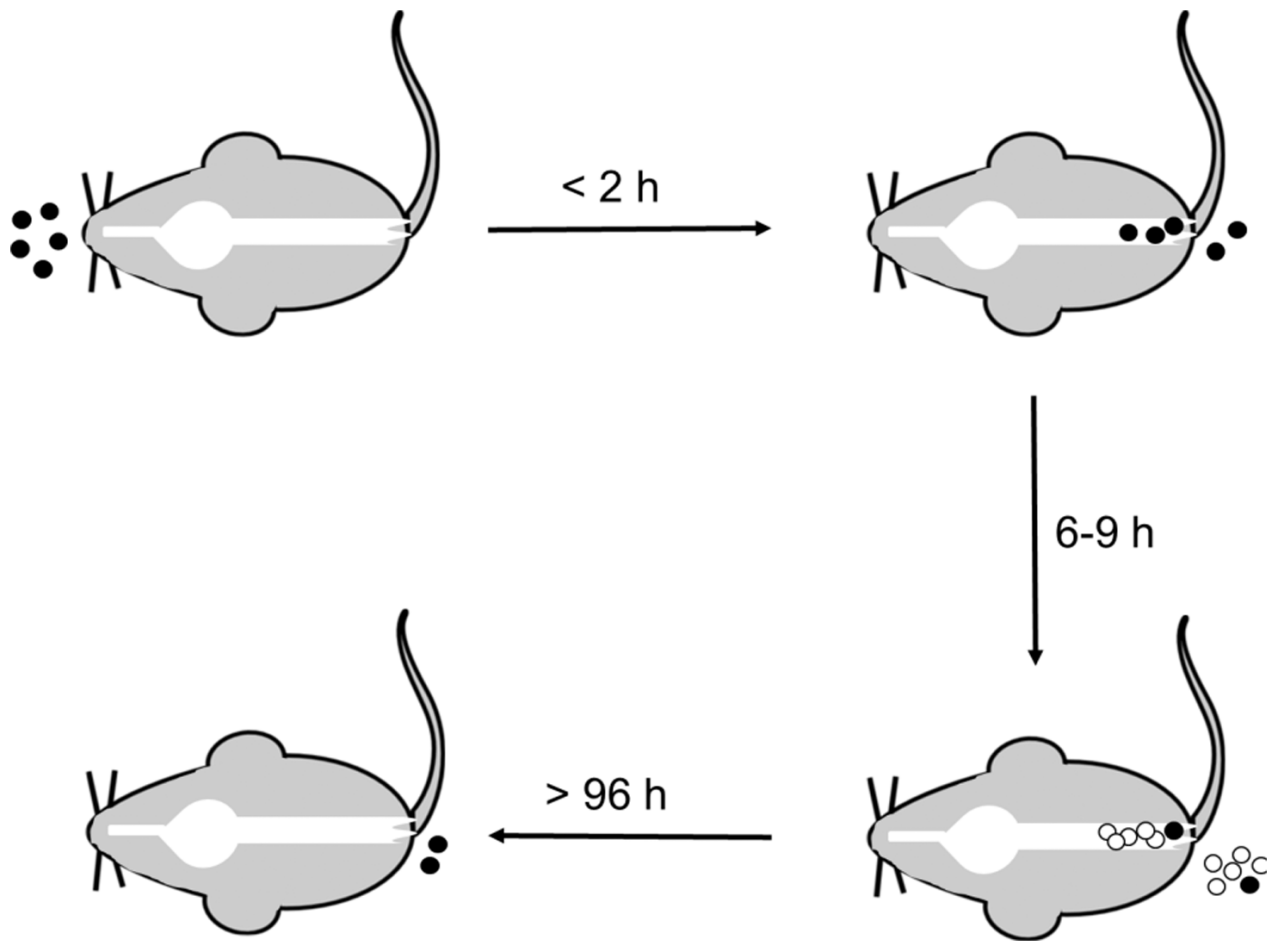
Time line model for CDI onset in mice. *C. difficile* spores (black circles) are ingested by the host. Spores rapidly transit through the upper GI tract and colonize the colon and cecum. Spore shedding begins less than 2 hours post-ingestion. Between 6 and 9 hours after ingestion sufficient numbers of spores germinate to establish infection. The outgrowing *C. difficile* cells (white circles) proliferate in the lower intestine, are shed, and can re-sporulate. A small amount of ingested spores remain in the lower intestine for more than 96 hours post ingestion.

CamSA’s anti-germination activity can be used to address mechanistic details about CDI initiation (Fig. 7). The sum of our data suggests that ingested spores rapidly transit through the GI tract (Fig. 6B) and accumulate in the lower intestine (Fig. 6A and Fig. S6). Six to nine hours after ingestion enough *C. difficile* spores germinate to establish infection (Fig. 5A). *C. difficile* vegetative cells start shedding almost immediately after germination and continue throughout the infection (Fig. 5C). In contrast, dormant *C. difficile* spores are slowly shed over a four day period (Fig. 6B). The timing of *C. difficile* spore germination and the persistence of ungerminated spores in the lower intestine can have profound implication in the prophylactic treatment of CDI.

## Materials and Methods

### Materials

Bile salts were purchased from Sigma-Aldrich Corporation (St. Louis, MO) or were synthesized in the Abel-Santos laboratory [8]. All bile salts were dissolved in DMSO prior to use. Artificial gastric juice, intestinal juice and PRO disks were purchased from Fisher Scientific (Pittsburg, PA). Thin layer chromatography (TLC) silica gel 60 F_254_ plates were purchased from EMD Chemicals (Gibbstown, NJ). Cell Titer-Glo luminescent cell viability assay kit was obtained from Promega Corporation (Madison, WI). *Clostridium difficile* selective agar plates were purchased from BD Biosciences (Franklin, Lakes, NJ). All bacterial strains used in this study were purchased from ATCC (Manassas, VA) and grown as suggested.

### Animals

This study was performed in strict accordance with the recommendations in the Guide for the Care and Use of Laboratory Animals of the National Institutes of Health. The protocol was reviewed and approved by the Institutional Animal Care and Use Committee at the University of Nevada, Las Vegas (Permit Number: R0411-266). Weaned Female C57BL/6N mice were purchased from Harlan laboratories (Indianapolis, IN). Animals were housed in groups of 5 mice per cage in the University of Nevada, Las Vegas animal care facility. All cages, water, food and bedding were autoclaved prior to contact with animals. Upon arrival, mice were allowed to acclimate for one week prior to experimentation. Post-challenge animal manipulations were performed in a biosafety level 2 laminar flow hood.

### Acute Toxicity of CamSA in Mice

To determine acute toxicity of CamSA, we used the Fixed Dose Procedure (FDP) [15]. CamSA was dissolved in DMSO to a concentration of 100 mg/ml. Groups of five mice were treated by oral gavage for three consecutive days with 50 mg/kg body weight of CamSA or chenodeoxycholate. A control group was administered DMSO. Weight changes were recorded daily and mice were observed for adverse reactions such as vomiting, diarrhea, hair loss, hunched posture, weight loss, and lethargy. Other groups of mice were treated with 300 mg/kg CamSA or chenodeoxycholate and observed as above.

### Prevention of CDI by Bile Salt Analogs

*C. difficile* 630 (ATCC BAA-1382) were prepared as previously described [11]. Purified *C. difficile* spores of 630 strain were used to challenge mice as published [16], [26]. Briefly, an antibiotic cocktail containing kanamycin (0.4 mg/ml), gentamycin (0.035 mg/ml), colistin (850 U/ml), metronidazole (0.215 mg/ml), and vancomycin (0.045 mg/ml) was prepared in autoclaved water and sterile filtered. For three consecutive days, mice were allowed to drink the antibiotic cocktail *ad libitum*. The antibiotic water was refreshed daily. After three days of antibiotic water, all mice received autoclaved water for the remainder of the experiment. A single dose of clindamycin (10 mg/kg) was administered by intraperitoneal (IP) injection on the fourth day (24 hours before *C. difficile* challenge). At this time, groups of five antibiotic-treated mice received neat DMSO, 300 mg/kg CamSA, 50 mg/kg chenodeoxycholate, 300 mg/kg taurocholate, or 300 mg/kg ethylcholate oral gavage. The day of challenge, animals received 10^8^ CFUs of *C. difficile* spores by oral gavage. One hour post challenge, animals received a second dose of the corresponding bile salt or DMSO. A third dose of bile salt or DMSO was administered 24 hours post-challenge. All animals were observed twice daily for signs of CDI. Disease signs were scored using the following rubric: pink anogenital area (score of 1), red anogenital area (score of 2), lethargy (score of 1), diarrhea/increase in soiled bedding (score of 1), wet tail (score of 2), hunchback posture (score of 2), 8-15% loss of body weight (score of 1), >15% loss of body weight (score of 2). Animals scoring 2 or less were undistinguishable from non-infected controls and were considered non-diseased. Animals scoring 3–4 were considered to have mild CDI with signs consisting of pink anogenital area, lethargy, an increase of soiled bedding and minor weight loss. Animals scoring 5–6 were considered to have moderate CDI with signs consisting of mild CDI signs plus red anogenital area and hunchback posture. Animals scoring >6 were considered to have severe CDI and were immediately euthanized. These animals displayed signs described above plus wet tail and severe weight loss. Asymptomatic animals were monitored for up to 14 days post-challenge to monitor CDI onset delay.

### Stability of CamSA in Artificial Gastric and Intestinal Juices

CamSA was analyzed for stability in simulated gastric and intestinal juices as published [27]. Briefly, 100 mg CamSA was added to 1 ml of either artificial intestinal or artificial gastric juice and incubated at 37°C. Aliquots were taken at 4, 8, 12, and 24 hours. Samples (1 µl) were spotted on silica TLC plates and allowed to air dry. Plates were developed with 75% ethyl acetate/methanol. TLC plates were visualized by spraying with a 10% wt/vol phosphomolybdic acid (PMA)/ethanol solution followed by heating at 100°C for 2 minutes. Quantification of CamSA was determined using a GE Healthcare Typhoon 9410 Variable Mode Imager and analyzed using ImageQuant TL 5.2 software.

### Stability of CamSA after Incubation with Bile Salt Hydrolase (BSH) Producing Bacteria

Following previous procedures [28], *Escherichia coli DH5α*, *Bifidobacterium longum* (ATCC BAA-999) and *Lactobacillus gasseri* (ATCC 33323) were incubated for 24 hours at 37°C. Bacterial cultures were then adjusted to an optical density of 1.0 with fresh media supplemented with 6 mM CamSA or taurocholate and incubated at 37°C. Samples of spent media were taken at 4, 8, 12, and 24 hours. Bile salt concentration was monitored by TLC as above. Percent conjugated bile salts were derived by dividing the intensity of TLC spots obtained at different times by the intensity of the TLC spot obtained before incubation.

### *In vitro* Permeability Assays

Caco-2 permeability assays of CamSA were performed by Apredica, LLC (Watertown, MA). Briefly, CamSA was dissolved in DMSO and added to Caco-2 cell cultures to 10 µM final concentration. CamSA was analyzed for both apical to basolateral permeability and basolateral to apical permeability across a Caco-2 cell monolayer. After a 2 hour incubation, CamSA concentrations in the apical and basolateral sides of the Caco-2 monolayers were determined by HPLC-MS. An *in vitro* ADME-Tox test was also conducted to estimate the percent recovery of CamSA from either the apical to basolateral permeability or basolateral to apical permeability.

### Effect of CamSA on Bacterial Growth

Laboratory strains of *E. coli DH5α*, *B. longum*, *L. gasseri*, and *C. difficile* were individually inoculated from freezer stock onto appropriate agar medium as directed by ATCC. Plates were incubated overnight at 37°C either aerobically (*L. gasseri* and *E. coli*) or anaerobically (*C. difficile* and *B. longum*). Anaerobic conditions consisted of a 5% CO_2_, 10% H_2_, and 85% N_2_ environment. Single cell clones were carefully selected and used to inoculate 5 mL of liquid medium. Inoculated broth was shaken at 37°C for approximately four hours until optical density at 580 nm reached 0.8 representing exponential phase of growth. Bacteria were sub-cultured (1∶100) into fresh media and individually supplemented with 10 mM CamSA or taurocholate. An increase of optical density at 580 nm (OD_580_) was used to measure exponential bacterial growth. The OD_580_ was recorded at 0, 1, 2, 3, 4, 6, and 8 hours post subculture inoculation. Growth inhibition was determined by comparing optical density of bile salt-treated cultures with untreated control cultures.

### Cytotoxicity of CamSA

Vero cells, Caco-2 cells, and murine macrophages J774A.1 were seeded in complete medium (minimum essential medium (MEM), 10% fetal bovine serum (FBS), and 1% penicillin/streptomycin). Cells were grown at 37°C with 5% CO_2_. Cells were detached by incubation with 1 mM EDTA-trypsin for 5 minutes. Complete medium was then added and monolayers lifted with a cell scraper. Cells were recovered at 800×g for 5 minutes at room temperature and the cell pellet was resuspended in fresh complete medium. A sample of cell suspension was treated with trypan blue to determine background non-viable cells [29]. Culture cells were plated in 12-well or 96-well tissue culture plates at a density of 10^5^ cells/ml and allowed to attach overnight. Spent medium was removed and fresh complete medium supplemented with 50 or 200 µM CamSA was added. As negative control, cells were treated with complete media supplemented with DMSO. As positive control, cells were treated with complete media supplemented with 10% EtOH. Plates were incubated overnight as described above.

The 12-well plates containing cell cultures were used as a qualitative method to determine cytotoxicity by visual observation of morphological changes, such as cell rounding. Cell viability was also determined by trypan blue dye exclusion staining. The 96-well plates cultured with mammalian cells were used as a quantitative method to determine cytotoxicity using the CellTiter Glo Luminescent cell viability assay. This assay quantitates the concentration of ATP, which indicates metabolically active cells [30]. After overnight CamSA-treatment, the 96-well plates were equilibrated to room temperature for 30 minutes before addition of the CellTiter-Glo reagent. Luminescence was read with an integration time of 1 second per well using a Tecan Infinite 200 plate reader and iControl software. The luminescence signals from cell cultures supplemented with DMSO only were set as 100% cell viability. Percent survival for other conditions was calculated relative to these untreated cells.

### *C. difficile* Toxin-induced Cell Death

*C. difficile* strain 630 spores were washed five times with nanopure water, heat activated at 68°C for 30 minutes and washed five more times with water. Spore pellets were resuspended in 0.1 M sodium phosphate buffer supplemented with 0.5% sodium bicarbonate (pH 6.0) to an OD_580_ of 1.0. Spores were diluted five-fold in BHI broth supplemented with 6 mM taurocholate/12 mM glycine (germination medium), germination medium supplemented with 50 µM CamSA, or germination medium supplemented with 200 µM CamSA. Spore suspensions were incubated anaerobically at 37°C overnight. The following day, *C. difficile* cells and spores were removed by centrifugation and spent media filter sterilized. In parallel, Vero and Caco-2 cells were cultured in 96-well plates, as described above. The mammalian cell cultures were then exposed to varying concentrations of sterile spent media for 24 hours. Cell viability was determined as before with the CellTiter Glo viability kit. The luminescence signals from cell cultures supplemented with 0% spent media were set as 100% cell viability. Percent survival for other conditions was calculated relative to these untreated cells.

### Onset of CDI Signs in Mice

Antibiotic treated mice were challenged by oral gavage with 10^8^ CFUs of *C. difficile* strain 630 spores. Individual groups of five mice were treated with a single 300 mg/kg dose of CamSA at 0, 6, 9 or 12 hours post-spore challenge. A second 300 mg/kg dose of CamSA was administered 24 hours after the first dose. Mice were observed for signs of CDI twice daily and scored accordingly.

### Enumeration of *C. difficile* Vegetative Cells and Spores

Spore challenged animals were treated with 0 or 300 mg/kg CamSA. Cages from infected animals were changed and feces were collected at different time points. Selected animals were sacrificed at different time points and their GI tract observed for signs of disease. Gastrointestinal tracts were removed in blocks and GI tract contents were flushed with autoclaved water. Feces and intestinal contents were weighed and homogenized in autoclaved water. Aliquots of the fecal suspensions were heated to 68°C for 30 minutes. Heated and unheated feces and GI tract contents were serially diluted in water and plated on *Clostridium difficile* selective agar (CDSA). Plates were incubated anaerobically for 48 hours and colonies were counted to enumerate colony-forming units (CFUs). CFUs obtained from unheated samples represent the sum of *C. difficile* vegetative cells and spores. CFUs obtained from heated samples represent the number of *C. difficile* spores only. The presence of *C. difficile* was verified by PRO disk.

### Statistical Analysis

Standard deviations represent at least three independent measures, unless otherwise stated. Recovered CFU and recovered spores represent mean values from a pool of five animals. Student's unpaired *t*-test was used to determine the significance of difference of means.

## Supporting Information

Figure S1 Mice treated with CamSA or chenodeoxycholate shows no weight changes. Groups of five mice were treated with DMSO (□), 50 mg/kg chenodeoxycholate (◊), 50 mg/kg CamSA (Δ), or 300 mg/kg CamSA (○). Animal weight was obtained daily.
(TIFF)

Figure S2 Protection of mice from CDI by different bile salts. Kaplan-Meier survival plot for *C. difficile* infected mice treated with DMSO (◊), 300 mg/kg taurocholate (Δ), 50 mg/kg chenodeoxycholate (○), 50 mg/kg CamSA (♦), or 300 mg/kg ethylcholate (×). Non-infected animals were used as control (□).
(TIFF)

Figure S3 Figure 3. Signs severity for *C. difficile* infected animals treated with different bile salts. Non-infected animals were used as control (panel A). Animals challenged with *C. difficile* spores were treated with three doses of DMSO (panel B), taurocholate (panel C), chenodeoxycholate (panel D), CamSA (panel E), or ethylcholate (panel F). The severity of CDI signs was scored using the Rubicon scale discussed above.
(TIF)

Figure S4 CamSA does not affect vegetative bacterial growth. *E. coli DH5α* (*□*), *B. longum* (○), *L. gasseri* (Δ), and *C. difficile* (◊) were incubated in media supplemented with 0 or 10 mM CamSA. The OD_580_ was recorded at 0, 1, 2, 3, 4, 6, and 8 hours. Growth inhibition was determined by subtracting optical density of CamSA-treated cultures from untreated control cultures.
(TIFF)

Figure S5 CamSA is not toxic to mammalian cells. Murine macrophages J774A.1 were treated with DMSO (panel A), 10% ethanol (panel B), or 200 µM CamSA (panel C). Cell viability was determined by trypan blue dye exclusion staining
(TIFF)

Figure S6 Distribution of C. difficile spores in the GI tract of CamSA-treated animals. The stomach (St), duodenum (Du), jejunum (Je), and ileum (Il) showed negligible amounts of spores compared to the cecum (Ce) and colon (Co).
(TIFF)

Table S1 CamSA permeability across Caco-2 cell monolayer^a^.
(DOCX)
