# Supplementary figures and images for: Fate of Ingested Clostridium difficile Spores in Mice
Source: PLoS One. 2013 Aug 30;8(8):e72620. doi: 10.1371/journal.pone.0072620 (PMC3758320; doi:10.1371/journal.pone.0072620)

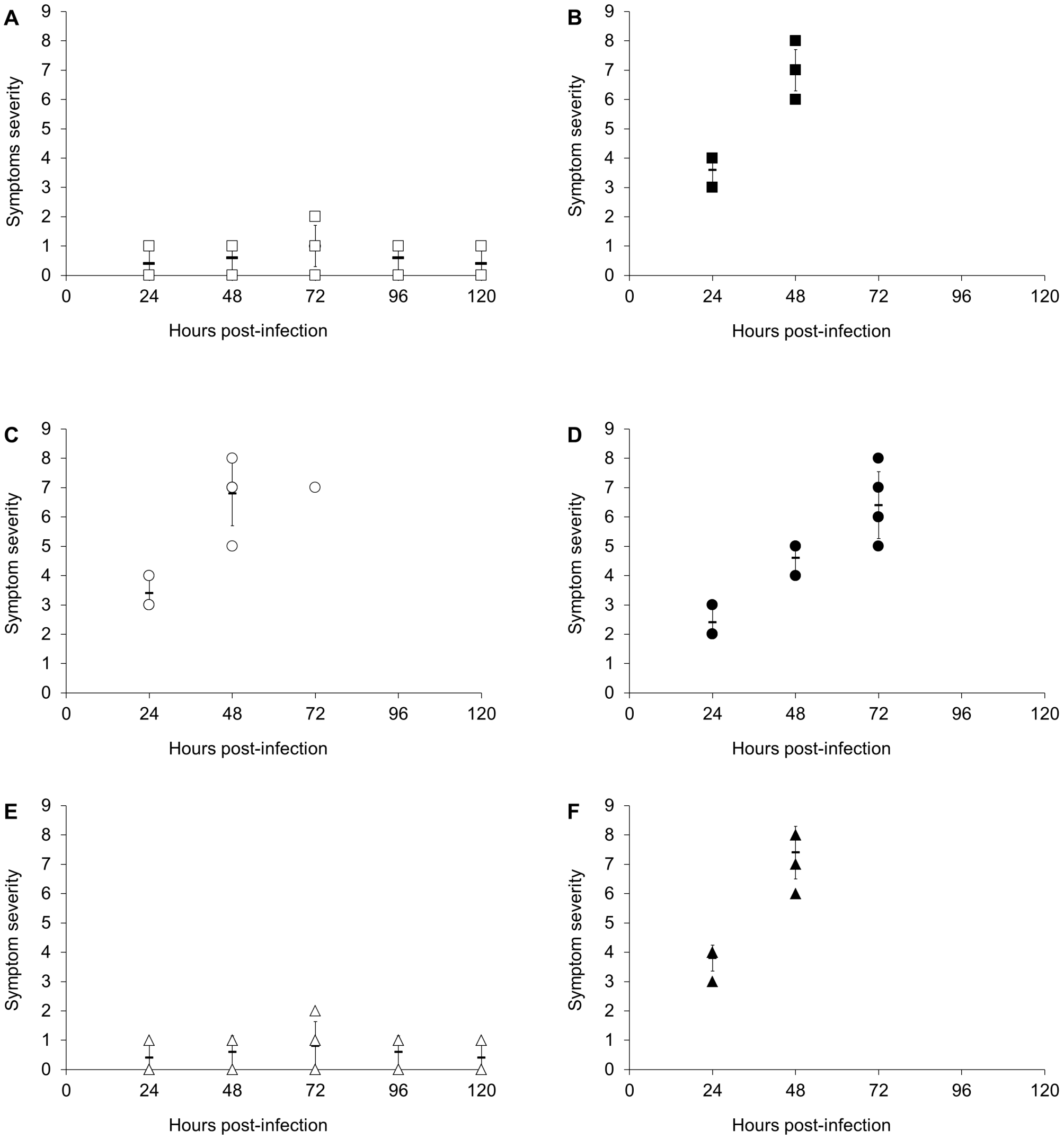

Supplement: Figure S3 — Figure 3. Signs severity for C. difficile infected animals treated with different bile salts. Non-infected animals were used as control (panel A). Animals challenged with C. difficile spores were treated with three doses of DMSO (panel B), taurocholate (panel C), chenodeoxycholate (panel D), CamSA (panel E), or ethylcholate (panel F). The severity of CDI signs was scored using the Rubicon scale discussed above. (TIF) [file pone.0072620.s003.tif]

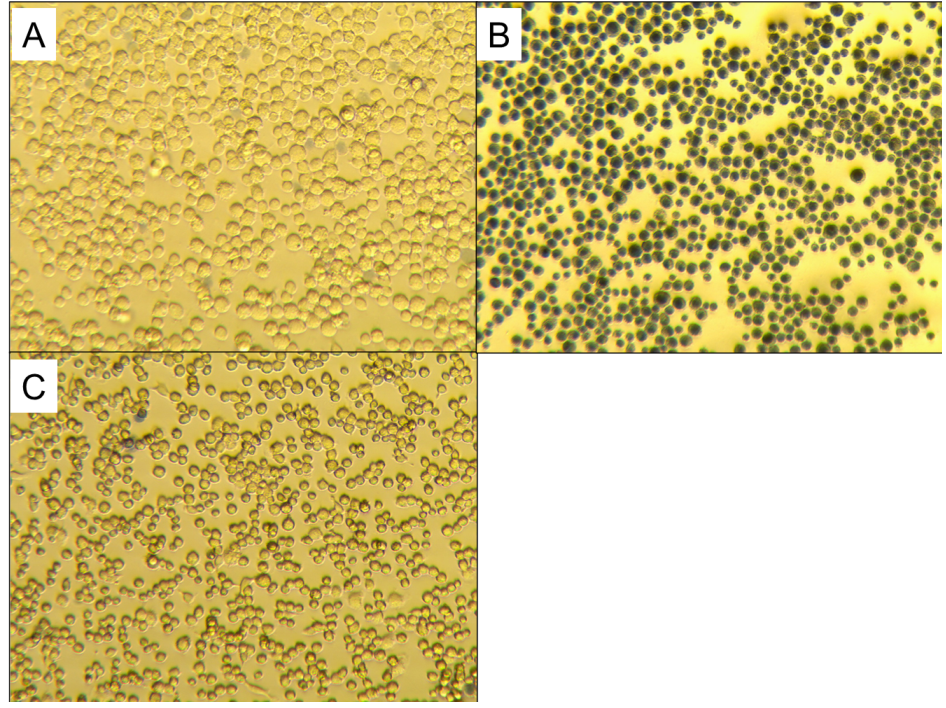

Supplement: Figure S5 — CamSA is not toxic to mammalian cells. Murine macrophages J774A.1 were treated with DMSO (panel A), 10% ethanol (panel B), or 200 µM CamSA (panel C). Cell viability was determined by trypan blue dye exclusion staining (TIFF) [file pone.0072620.s005.tiff]
