# Supplementary material for: Fate of Ingested Clostridium difficile Spores in Mice
Source: PLoS One. 2013 Aug 30;8(8):e72620. doi: 10.1371/journal.pone.0072620 (PMC3758320; doi:10.1371/journal.pone.0072620)
Supplement: Table S1 — CamSA permeability across Caco-2 cell monolayera. (DOCX) [file pone.0072620.s007.docx]

| **Table S1. CamSA permeability across Caco-2 cell monolayer^a^** | | | | |
| --- | --- | --- | --- | --- |
| **Compound** | **A→B^b^**  **(10^-6^ cm/s)** | **B→A^c^**  **(10^-6^ cm/s)** | **Efflux Ratio**^d^ | **Comment^e^** |
| Ranitidine | 0.3 | 1.8 | 5.3 | Low permeability control |
| Warfarin | 42.9 | 16.0 | 0.4 | High permeability control |
| CamSA | 0.0 | 10.9 | >2 | Low Permeability. Efflux substrate |
| ^a^ All tests were performed at 10 µM final concentrations and equilibrated for two hours  ^b^ Apical to basolateral apparent permeability (P_app_)  ^c^ Basolateral to apical apparent permeability (P_app_)  ^d^ Efflux ratio (RE) >2 indicates a significant efflux activity, an indication of potential substrate for PGP or other active transporters  ^e^ Permeability ranking: Low (P_app_ < 0.5), Moderate (0.5 < P_app_ < 5), High (P_app_ > 5) | | | | |
